# Elucidating the Intriguing Association Between Systemic Lupus Erythematosus and Cardiovascular Disease

**DOI:** 10.7759/cureus.15538

**Published:** 2021-06-09

**Authors:** Mushrin Malik, Rajvi Gor, Nabeel A Siddiqui, Dhairya Gor, Kazi I Ahmed

**Affiliations:** 1 Research, California Institute of Behavioral Neurosciences & Psychology, Los Angeles, USA; 2 Research, California Institute of Behavioral Neurosciences & Psychology, Fairfield, USA; 3 Internal Medicine, Jersey Shore University Medical Center, Neptune, USA; 4 Internal Medicine, University of Dhaka, Dhaka, BGD

**Keywords:** sle, autoantibodies, autoimmune vasculitis, atherosclerosis, cvd & sle

## Abstract

Systemic lupus erythematosus (SLE) patients have demonstrated a higher risk of developing cardiovascular disease (CVD), resulting in it being one of the leading causes of death in SLE patients. SLE itself acts as a sole risk factor influencing the prevalence and progression of CVD. However, conventional risk factors, such as age, hypertension, smoking, and obesity, play a crucial role as well. Therefore, this systematic review attempts to unravel the association of CVD in SLE patients while evaluating the role of conventional risk factors.

Preferred Reporting Items for Systematic Reviews and Meta-Analyses (PRISMA) guidelines were followed to search the PubMed database starting from March 2021 systematically. Original studies that evaluated the prevalence and progression of CVD in SLE patients were extracted by two reviewers independently. Quality in Prognostic Studies (QUIPS) tool was used to assess the risk of bias. Most studies have a moderate to low risk of bias. Among 3,653 studies identified by our search, 10 studies were included in the review. Strong epidemiologic evidence of SLE patients having an increased relative risk of CVD compared to controls was found. Traditional CVD risk factors, such as age, hypertension, obesity, and smoking, influence the prevalence of CVD among SLE patients. Several SLE-specific factors such disease activity, duration, and certain medications also acted as influencing factors. However, the relative risk of CVD was still higher in SLE patients after adjustment of certain risk factors. One study found that the odds of having a Coronary Artery Calcification (CAC) score greater than zero in women with SLE aged less than or equal to 45 years was 12.6 times higher than women in the Coronary Artery Risk Development in Young Adults (CARDIA) cohort (95% CI 5.2 to 30.7) (participants of CARDIA cohort acted as control). This finding was made after age, hypertension, total cholesterol levels, and aspirin use were adjusted, and the study was restricted to women.

Although conventional risk factors increase CVD prevalence, SLE itself also dramatically increases the prevalence of CVD. Therefore, we recommend that SLE should be treated as a "CVD risk equivalent." SLE patients should be managed more extensively with greater emphasis given to cardiac health for better clinical outcomes.

## Introduction and background

Cardiovascular disease (CVD) is the leading cause of morbidity and mortality, both in developed and developing countries. The underlying pathological change in CVD is atherosclerosis. Endothelial cell injury initiates atherosclerosis with deposition of oxidized low-density lipoprotein (oxLDL) in the arterial wall resulting in the activation of monocytes, which then get attracted to the subendothelial space and become activated macrophages. Macrophages then internalize oxLDL from the circulation and arterial smooth muscle cells, forming a lipid-rich cell called “foam cells.” A variety of cytokines are produced by macrophages which accelerate smooth muscle cell and fibroblast migration. As a result, a plaque inside the vascular wall, also known as an atheroma, is formed [1].

Systemic lupus erythematosus (SLE) is a chronic, autoimmune disorder with a variable clinical course involving multiple body organs. SLE affects 20-150 per 100,000 individuals, with most cases (70%-90%) occurring in women [2]. Common complications of SLE that result in significant morbidity and mortality include infection, nephritis, stroke, peripheral artery disease (PAD), and CVD [3,4]. In 1976, Urowitz et al. described the bimodal mortality pattern in SLE patients, which stated that death within the first three years after diagnosis was usually due to active disease, infections, and glomerulonephritis. Death later in the disease course, almost four to 20 years after SLE diagnosis, was usually due to CVD [5]. This hypothesis resulted in a growing interest in both the epidemiology and pathophysiology of CVD among SLE patients. 

Multiple risk factors, such as hypertension, hyperlipidemia, and smoking, have been shown to predict and influence CVD onset in patients with SLE. However, multiple pathophysiological processes in SLE itself are an independent risk factor for CVD [6]. Mechanisms by which SLE itself promotes arterial wall injury include renal disease, hypertension, antiphospholipid antibodies, thrombosis, treatment with corticosteroids, and the endothelial response immune-complex mediated inflammation [7].

With advances in treatment and a better understanding of disease mechanisms, overall mortality for patients with SLE has improved in the last 30 years. However, deaths due to CVD in SLE patients have remained the same [8]. Therefore, for better clinical outcomes, we must determine the prevalence and progression of CVD in SLE patients and ways to predict the risk of developing CVD in SLE patients to identify patients who would require a more aggressive treatment approach.

In this review, we look at the connection between SLE and CVD. We aim to highlight the prevalence of CVD and understand CVD progression in SLE patients with respect to other CVD risk factors.

## Review

Method

Search Strategy and Selection Criteria

The following medical topic heading terms and keywords were used to search the PubMed database: SLE, autoantibodies, autoimmune vasculitis, atherosclerosis, CVD, and SLE. The search protocol for our systematic review was based on Preferred Reporting Items for Systematic Reviews and Meta-Analyses (PRISMA) Group Guidelines, starting from March 2021 [9]. Only original studies on human subjects published in the English language were included in the search. Two reviewers independently performed the screening process to acknowledge all citations of possible acceptability, including searching the reference list of pertinent articles for additional sources. We excluded review articles, nonhuman studies, case reports, letters, conference abstracts, and editorials. Basic science studies concerning the mechanism of atherosclerotic disease and studies related to non-atherosclerotic CVD, such as pericarditis, myocarditis, conduction system disease, and valvular disease, were also excluded. Articles that targeted only the antiphospholipid antibody syndrome without concomitant SLE were in the exclusion criteria as well. Our search was limited to studies that examined the prevalence and progression of CVD in SLE patients. Kappa statistics were used to evaluate the inter-rater reliability of the two reviewers [10].

Data Extraction

All abstracts were screened, and eligibility criteria were applied to identify studies that were appropriate for inclusion. Data were then extracted independently using predetermined criteria, including date of publication, population, language, study design, duration, participant data, outcome definition, results, and risk of bias. 

Methodological Quality Assessment

Two independent investigators evaluated the possibility of bias in the included studies using the Quality in Prognosis Studies (QUIPS) method developed by Hayden et al. [10] to evaluate each article. The QUIPS tool employs 30 parameters divided into six domains (patient selection, study attrition, prognostic factor calculation, result measurement, confounding measurement and account, and statistical analysis and reporting). Each criterion is scored as “yes,” “no,” or “unclear.” Therefore, each domain is evaluated as being of “low,” “moderate,” or “high” risk of bias according to the scoring system. A study was considered high quality when the bias was scored as low or moderate concerning almost all domains. Conversely, a study was deemed low quality when the bias was rated high in most of the bias domains. Any disputes were resolved by consensus.

Results

Literature Search

**Figure 1:**
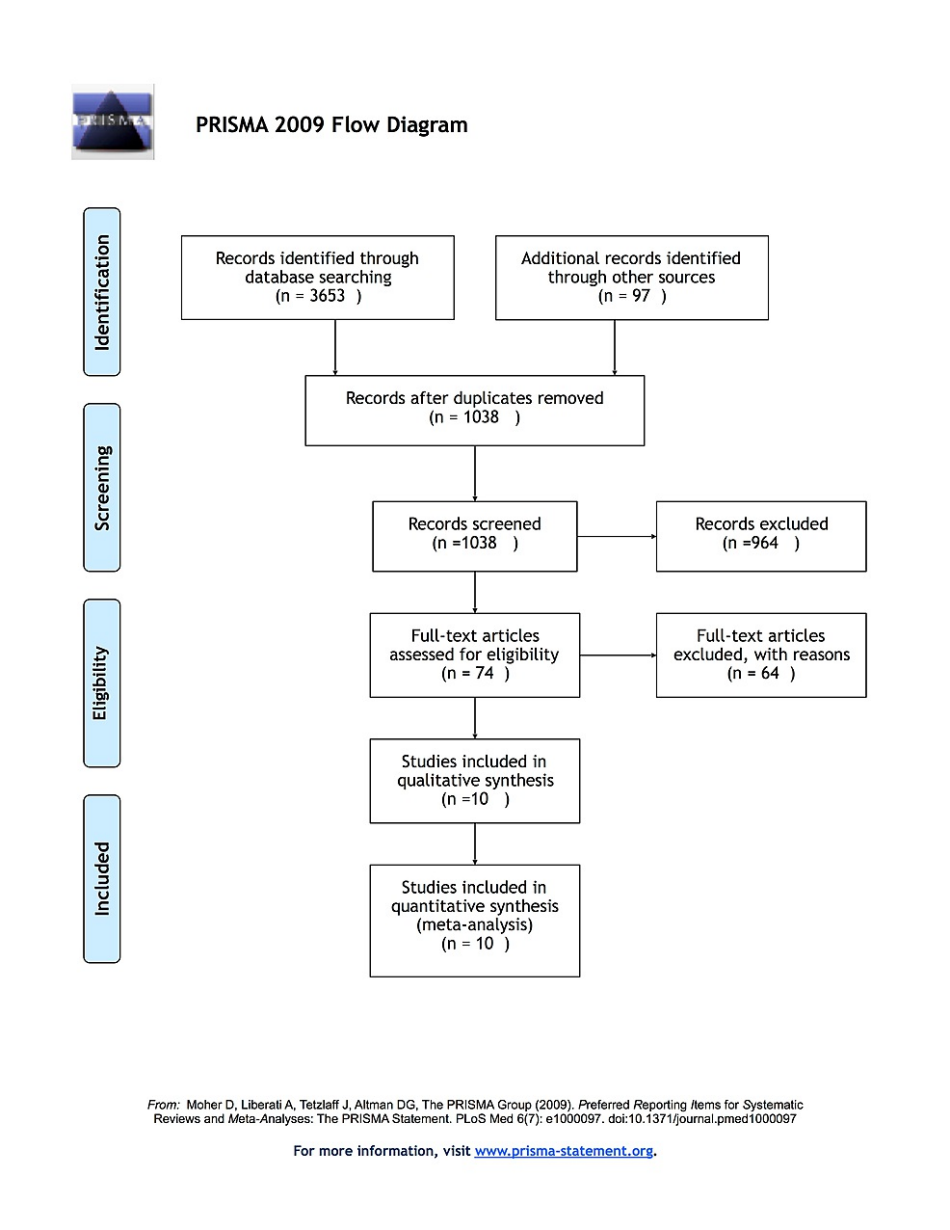
PRISMA flow diagram PRISMA, Preferred Reporting Items for Systematic Reviews and Meta-analyses

We identified ten studies through our screening process out of 3,653 citations that met the inclusion criteria of our analysis. Figure 1 portrays a flow diagram of study identification and eventual inclusion. A total of 891 patients participated in the study, and 420,142 clinical cases were analyzed from hospital records.

Characteristics of the Included Studies

Table 1 demonstrates the characteristics of included studies. In this review, we included studies that were published from 2011 to 2020 and the included studies were Gartshteyn et al., Levinson et al., Stojan et al., Ishimori et al., Kaul et al., Katz et al., Khan et al., Plazak et al., Kao et al., and Lertratanakul et al. [11-20]. Almost all studies (90% studies) were conducted in the United States of America [11-17,19,20]. Only one study (10%) was conducted in Poland [18]. Only observational and non-interventional studies were included in the review. Among the included studies, six studies were prospective cohort [11-14,19,20]. Two studies were longitudinal [17,19]. One study was a retrospective cohort [15]. One study was case-control [15]. The prevalence of CVD in SLE patients was predicted using multidetector computed tomography (MDCT) scan in three studies [11,18,20]. Coronary computed tomography angiography (CCTA) scan was used in three studies [13,14,17]. International Classification of Diseases, Ninth Revision (ICD-9) Codes were used to describe CVD in two studies [12,16]. Cardiac magnetic resonance (CMR) was used in one study [14]. A cardiac angiogram was done in one study [15]. Single-photon emission computed tomography (SPECT) was done in one study. B-mode carotid ultrasound was done in one study [19].

**Table 1:** Study Characteristics MDCT- Multidetector Computed Tomography CAC- Coronary Artery Calcification ICD-9 - International Classification of Diseases, Ninth Revision CCTA- Coronary Computed Tomography Angiography LANCP- Low Attenuation non-calcified plaque CMR- Cardiac Magnetic Resonance MRPI- Quantitative Myocardial Perfusion Reserve Index NCP- Non-Calcified Plaque SPECT- Single-photon emission Computed Tomography IMT- Intima Media Thickness

Study	Study Design	No. of Participants	Predictor used	Factor Evaluated
Gartshteyn et al. [11]	Prospective Cohort	76	MDCT scan	CAC
Levinson et al. [12]	Prospective Cohort	167,466	ICD-9 codes	-
Stojan et al. [13]	Prospective Cohort	72	CCTA scan	LANCP
Ishimori et al. [14]	Prospective Cohort	20	CCTA scan, CMR	CAC, MRPI
Kaul et al. [15]	Retrospective Cohort	86	Cardiac angiography	Obstructive CVD
Katz et al. [16]	Nested Case-Control	252,676	ICD-9 codes	-
Khan et al. [17]	Longitudanal Prospective	36	CCTA scan	NCP, CAC
Plazak et al. [18]	Cross-sectional Study	60	MDCT scan, SPECT scan	CAC
Kao et al. [19]	Longitudanal Prospective	392	B-mode carotid US	Carotid IMT and plaque
Lertratanakul et al. [20]	Prospective Cohort	149	MDCT scan	MDCT

Risk of Bias Assessment

**Figure 2:**
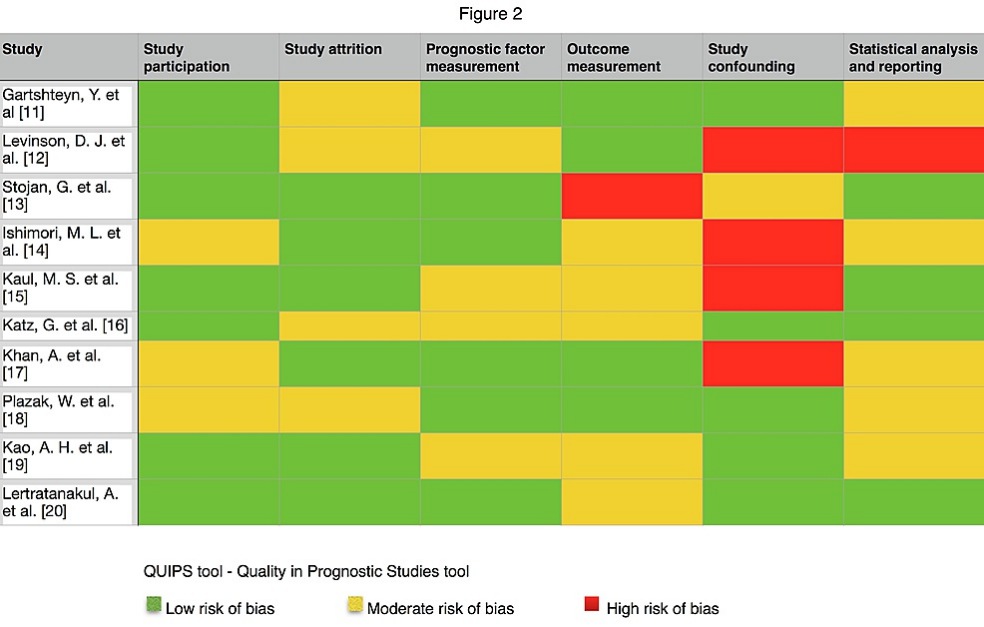
Risk of Bias Assessment QUIPS - Quality in Prognostic Studies

Figure 2 demonstrates the risk of bias assessment of the review. Most studies showed to have a low to moderate risk of bias. Five out of 10 studies showed a high risk of bias in at least one domain [11-14,16]. Moderate to high risk of bias in confounding measurement was found in five out of 10 studies [11-14,16]. One study did not mention confounders [13]. All 10 studies used statistical models to evaluate prognostic relationships in which five out of 10 studies showed a moderate risk of bias [10,13,16-18].

Predictors of CVD

Gartshteyn et al. used MDCT to assess the Coronary Artery Calcification (CAC) score (CAC score classification is in appendix) in SLE patients at two intervals [11]. Levinson et al. and Katz et al. used ICD-9 codes to predict the prevalence of CVD from the years 2010-2015 and 2008-2014, respectively [12,16]. Stojan et al. used CCTA to assess Low Attenuation non-calcified plaque (LANCP) (LANCP evaluation in appendix) having a follow-up period of one year [13]. Ishimori et al. used CMR to assess quantitative myocardial perfusion reserve index (MPRI) and CCTA to assess the CAC score [14]. A coronary angiogram was done to evaluate obstructive CAD by Kaul et al. [15]. Khan et al. used CCTA to assess the CAC score and non-calcified plaque in SLE patients at two and eight-year intervals [17]. Plazak et al. used MDCT and SPECT to determine the CAC score [18]. Kao et al. used B-mode carotid US to assess carotid intima media thickness (IMT) and plaque with a mean follow-up period of eight years [19]. Lertratanakul et al. used MDCT to assess CAC and aorta calcium (AC) [20].

Prevalence and Progression of CVD in SLE patients

Eight out of 10 studies evaluated the prevalence of CVD in SLE patients [11-16,18,19]. Two studies assessed the progression of CVD in SLE patients [17,20]. Gartshteyn et al. had 76 patients and the Coronary Artery Risk Development in Young Adults (CARDIA) cohort participants, aged 33-45, acted as controls [11]. CAC>0 was found in 9.6% of CARDIA participants, as well as 29.0%, 42.1%, and 61.6% of SLE patients aged 18-32, 33-45, and 46-64, respectively. CAC scores 1-99 vs. 100 were seen in 8.0% and 1.6% of CARDIA participants, respectively, and 29.0%, 26.3%, and 38.5% and 0%, 15.8%, and 23.1% of SLE patients aged 18-32, 33-45, and 46-64. Sex, race, and smoking status were not adjusted due to the large patient population. The odds of having a CAC>0 in women with SLE aged ≤45 was 12.6 times higher than those in women in the CARDIA cohort (95% CI 5.2 to 30.7) after the study was restricted to women and age, hypertension, total cholesterol levels, and aspirin use were adjusted.

Levinson et al. evaluated hospital records of 167,466 SLE patients using ICD-9 codes, and the prevalence of CVD in SLE patients was 17.2% compared to 14.5% in controls [12]. Stojan et al. enrolled 72 cases and 100 controls ( no known history of lupus, heart disease, or revascularization ) for evaluation using two CCTA scans one year apart [13]. Patients with SLE have a significantly more significant LANCP burden (p<0.001), although the control group predominantly included the male population. A substantially more significant LANCP burden was noticed in middle-aged women with SLE (age 45-59) compared with controls. There was no statistically significant variation between patients aged 60 and over. The control group included women older than 45, and the number of male patients with SLE was small.

Ishimori et al. included 20 cases and 10 controls (without evidence of CVD) and used CCTA to assess CAC and CMR to assess MRPI [14]. Only two SLE patients had mild coronary atherosclerosis (isolated non-calcified plaque with 25% to 49% stenosis in the left anterior descending coronary artery in one patient and a CAC score of 5.9 in another patient, consistent with minimal calcification), but no patient had obstructive CVD. On CMR, stress-induced hypoperfusion was found in eight of 18 (44%; 95% confidence interval: 21.5% to 67.4%) SLE patients compared to zero of ten of the reference control group (Fisher exact test p-value = 0.014) by semiquantitative visual analysis. The average MPRI in patients versus controls was 2.0 ± 0.4 versus 2.3 ± 0.4 (p = 0.16); 2.0 ± 0.4 versus 2.4 ± 0.4 in the subepicardium (p = 0.031) and 1.8 ± 0.3 versus 2.1 ± 0.4 in the subendocardium (p = 0.24).

Eighty-six cases and 258 controls (randomly selected and matched by sex and year of catheterization) were assessed in the study conducted by Kaul et al., where coronary angiography was performed to evaluate the prevalence of CVD [15]. On angiogram, 45 (52%) of SLE cases and 160 (62%) controls had obstructive CVD (p-value=0.11), which was not statistically significant. SLE was not associated with increased risk when covariate adjustment in the logistic regression model was not made, although SLE was significantly related to the presence of CVD on coronary angiograms after adjustment for traditional risk factors.

Katz et al. also used ICD-9 codes to assess CVD prevalence, having 758,034 controls (people without SLE) and 252,676 cases [16]. A higher prevalence of ASCVD was found in SLE patients compared with control patients (25.6% vs. 19.2%, OR 1.45, 95% CI 1.44 to 1.47, P<0.001), observed in both women (OR 1.47, 95% CI 1.45 to 1.48) and men (OR 1.40, 95% CI 1.36-1.44). In younger individuals, the association between SLE and ASCVD was most evident. The greatest odds of ASCVD were found in men (OR 4.05, 95% CI 3.33-4.93) and women (OR 12.44, 95% CI 11.13-13.91) aged 20-29 with SLE in comparison to age and sex-matched controls. On the contrary, men (OR 1.25, 95% CI 1.18-1.34) and women (OR 1.29 95% CI 1.26-1.32) aged 60-69 with SLE had only slightly increased odds of ASCVD.

Khan et al. evaluated 36 patients using CCTA to assess CAC and non-calcified plaque (NCP) progression over two to eight years [17]. NCP was seen in 24 of 36 (75%) of the SLE patients at the first assessment. However, only 12/35 (34%) had evidence of calcified plaque. Progression of NCP occurred in 12/36 (33%) patients, and 5/36 (14%) patients showed plaque regression, proving that NCP levels were variable for two to eight years. In 2/35 (5.71%) patients, progression of calcified coronary plaque was observed. Regression of calcified plaque was found in one out of 35 (2.85%) of patients, implying that calcified coronary plaque levels remained essentially constant over two to eight years.

Plazak et al. included 60 patients and used MDCT and SPECT to assess CAC scores [18]. Myocardial perfusion abnormalities were seen in 30 (50.0%) patients: persistent defects in 22 (36.7%) patients, exercise-induced defects in eight (13.3%) by doing SPECT study. Coronary calcifications were found in 15 (25%) patients using MDCT. 392 patients were assessed using B-mode carotid US to evaluate carotid IMT and plaque by Kao et al. with a mean follow-up period of eight years [19]. The study found that women with SLE had significantly higher mean carotid IMT at baseline (0.80 vs. 0.64 mm) and had an increased likelihood to have a carotid plaque at baseline (76.5% vs. 30.4%) compared with those without any incident hard CV events (all p-values <0.01).

Lertratanakul et al. enrolled 149 patients and 124 controls to evaluate the progression of CAD in SLE patients using MDCT to assess CAC and AC [20]. CAC progression at follow-up was found in 27 cases (18.1%) compared with 16 controls (12.9%). Progression in AC at follow-up was found in 32 cases (28.3%) compared with 22 controls (18.0%). In 112 SLE cases, where both AC and CAC were measured at baseline and at follow-up, 13 (11.6%) had progression in both compared to AC and CAC progression in 13 (10.7%) out of 122 controls.

Other Risk Factors That Affect the Prevalence of CVD in SLE

(i) Age: Seven out of ten studies evaluated the role of age affecting the prevalence and progression of CVD in SLE patients [11-13,15,16,19,20].

Gartshteyn et al. discovered that a CAC score of more than zero increased with age: CAC>0 was seen in 29.0%, 42.1%, and 61.6% of SLE patients aged 18-32, 33-44, and 45-64 years, respectively [11]. Although, patients with SLE were younger (32±8 vs. 40±4 years, p<0.001) compared to control.

Young patients with SLE had higher prevalence of chronic kidney disease (29.9% vs. 5.1%; SMD = 0.7), hypertension (45.9% vs. 13.2%; SMD = 0.8) and hypercoagulability (7.6% vs. 0.5%; SMD = 0.4) compared to controls which was observed by Levinson et al. [12]. As the population aged, the difference in the prevalence of these risk factors became less prominent. On the contrary, diabetes mellitus (DM) was less frequent in SLE versus controls in all age groups. Hyperlipidemia was less frequent in younger SLE patients with CVD than controls (SLE 21.6% vs. control 37.2% SMD = 0.4). It remained lower in older age groups with a moderate effect size.

Stojan et al. found LANCP to be associated with increasing age (p<0.05) [13].

Kaul et al. found that patients with SLE were significantly younger compared to controls (median age 49 years vs. 70 years, p<0.001) at the time of cardiac catheterization [15]. SLE had the greatest adjusted odds of ASCVD compared to matched control patients in younger women, according to Katz et al. [16]. 

Kao et al. found that patients with incident hard CV events were significantly older than patients without incident hard CV events [19]. Lertratanakul et al. found older age to be associated with CAC and AC progression in univariate models [20].

(ii) Hypertension and renal diseases: The effect of hypertension and renal diseases on CVD prevalence in SLE patients was evaluated in six out of 10 studies [11,12,16,18-20].

Gartshteyn et al. found SLE patients to have more comorbid hypertension (44% vs. 23%, p<0.001) [11].

Levinson et al. found that there was an increased prevalence of chronic kidney disease (29.9% vs. 5.1%; SMD = 0.7) and hypertension (45.9% vs. 13.2%; SMD = 0.8) in SLE patients compared to control group [12].

The finding that SLE patients were more likely to have hypertension and renal disease was observed by Katz et al. [16].

Plazak et al. found no influence of hypertension on coronary calcification formation or myocardial perfusion defects [18].

SLE patients with incident hard CV events have elevated systolic blood pressure than people without such hard CV events, according to Kao et al. [19]. 

Lertratanakul et al. observed that CAC progression is associated with lower GFR in univariate models and CAC and AC progression associated with lower GFR and hypertension in multivariate models [20].

(iii) Other CVD risk factors (smoking, hyperlipidemia, obesity, and DM): Five out of 10 studies evaluated the role of smoking, hyperlipidemia, obesity, and DM on CVD prevalence in SLE patients [11,12,16,18,19].

Gartshteyn et al. found that patients in the CARDIA cohort, i.e., the control group were more likely to smoke (4% vs. 56%, p<0.001) [11].

Levinson et al. found that DM is less prevalent in SLE versus controls in all age groups, and hyperlipidemia is less prevalent in younger SLE patients with CVD compared to controls (SLE 21.6% vs. control 37.2% SMD = 0.4) [12].

SLE patients were less likely to have hyperlipidemia, obesity, active tobacco use, or DM compared to control, according to Katz et al. [16]

Plazak et al. found that tobacco use, hyperlipidemia, obesity, and DM do not influence coronary calcification formation or myocardial perfusion defects [18]. Kao et al. found elevated total cholesterol levels associated with increased incident hard CV events [19].

A higher cholesterol/HDL ratio was associated with CAC progression in univariate models, according to Lertratanakul et al. [20].

(iv) Medication: Five out of 10 studies evaluated the roles of different medications on the prevalence and progression of CVD in SLE patients [11,13,17,19,20].

Gartshteyn et al. observed a higher aspirin use was found in SLE patients compared to control (32% vs. 7%, p<0.001) [11]. A daily dose of >10mg of prednisone was associated with LANCP, according to Stojan et al. [13].

Khan et al. found some evidence (p=0.06) of an association between immunosuppressant use and a minor progression of NCP [17].

Kao et al. found incident hard CV events to be more prevalent in patients taking lipid-lowering agents at baseline [19]. Lertratanakul et al. discovered that aspirin use was correlated with CAC progression of cases in univariate and multivariate models (OR 2.15, 95% CI 1.33-3.57 and OR 4.23, 95% CI 1.53-11.74, respectively), while corticosteroid use was associated with CAC progression only in multivariate models (OR 2.93, 95% CI 1.14-7.86) [20]. In controls, CAC progression was univariately found to be associated with aspirin use. The use of corticosteroid was also associated with AC progression (OR 2.73, 95% CI 1.03-7.64).

(v) SLE factors: Two out of 10 studies evaluated the role of SLE disease activity and antibodies in the prevalence and progression of CVD in SLE patients [19,20].

Plazak et al. found that inflammatory biomarkers like high CRP were not significantly associated with atherosclerotic lesions or perfusion disturbances [18]. However, regardless of their age, and increased anti cardiolipin (aCL) IgG and anti-β2-glycoprotein I (aβ2GPI) IgG levels were found in patients with atherosclerotic plaques in coronary vessels or with myocardial perfusion defects. Moreover, a significantly higher level of antinuclear antibodies and higher frequency of lupus anticoagulant (LA) incidence were observed in patients with coronary calcifications. Elevated levels of aCL IgG >20 RU/mL or antiβ2GPI IgG >3 RU/mL were associated with the relative risk of coronary calcification formation by 4.1 compared to patients with normal values. Consistently, the relative risk of coronary calcification formation in LA positive patients was 4.4 compared to LA-negative patients. 

Lertratanakul et al. found that a higher modified ACR/SLECC-DI score is associated with CAC progression in univariate models [20].

Discussion

The data collected in our review strongly supported that in patients with SLE, the prevalence of CVD is significantly higher, occurs in a younger age group, and is impacted by both conventional risk factors and the burden of immune-mediated inflammation [4,8,21-23]. The most significant relative risk was found in younger patients with SLE compared to their healthy counterparts. However, the absolute risk of CVD among SLE patients increased with advancing age.

In our review, eight out of 10 studies evaluated the prevalence of CVD in SLE patients. Two out of 10 studies assessed the progression of CVD in SLE patients. All the studies found a positive correlation between SLE and CVD. Furthermore, as most of the studies (six out of 10) were prospective cohorts, it helped understand the CVD clinical course in SLE patients better.

Traditional CVD risk factors, like hyperlipidemia, obesity, cigarette smoking, advancing age, hypertension, male sex, renal disease, DM, and elevated C-reactive protein, were all associated with increased CVD risk among SLE patients according to the collected epidemiological data. All of these risk factors were not examined simultaneously in the same populations. Therefore, the relative risk associated with each was not possible to evaluate, although we found the relative risk of CVD in SLE to be higher, even after adjusting certain risk factors. Gartshteyn et al. discovered that the chances of developing CAC>0 in women with SLE aged 45 years were 12.6 times higher than in women in the CARDIA cohort (95% CI 5.2 to 30.7) after age, hypertension, overall cholesterol levels, and aspirin usage were modified [11].

Several SLE-associated factors have also been predictive of CVD risk in the reviewed cohort studies in addition to conventional risk factors. These SLE-related factors include SLE disease duration, medications used, particular antibodies such as aCL IgG, antiβ2GPI IgG, and ACR/SLECC-DI score. The differences between the relative importance of risk factors for CVD among SLE patients in the reviewed studies is most probably due to differences in design methods and differences in patient and comparison groups. Moreover, the variability among these risk factors is considered simultaneously in multivariate models, making it challenging to separate inherently related elements. Sadly, certain SLE-related factors, such as disease activity, organ damage, and antiphospholipid antibodies, were not included in administrative data.

We also know that traditional stress tests can only detect flow-limiting stenosis and may miss early coronary atherosclerosis. Noninvasive imaging of coronary plaques is much more promising and superior resulting in significant advances in our understanding of atherosclerosis and its pathogenesis. Moreover, most of the studies we reviewed (eight out of 10) relied on noninvasive imaging techniques to detect the prevalence and progression of CVD in SLE patients, our understanding of the relation between CVD and SLE became more elaborate [11,13-15,17-20].

We must recognize shortcomings in our study, including the exclusion of non-translated non-English articles, inconsistency in the accuracy of primary research due to the presence of confounding variables, and the use of various methodological approaches by different studies in determining outcomes - these issues rendered incorporating findings in the analysis of results challenging.

## Conclusions

In conclusion, SLE is associated with higher prevalence and faster progression of CVD, making SLE patients particularly vulnerable, requiring more extensive coronary health management. Therefore, it can be proposed that SLE be treated as a "CVD equivalent” such as DM, with lower lipid goals, more aggressive aspirin use, and potentially more aggressive monitoring. However, we must conduct more randomized clinical trials to evaluate the effect of aggressive coronary care in patients with SLE.

## Appendices

CAC Scoring

CAC scoring ranges from 0 to 400. A Higher score correlates with a greater chance of an annual CVD event like myocardial infarction (MI). A score closer to 0 indicates lower chances of CVD.

LANCP

A LANCP score of <30 Hounsfield Units (HU) contains necrotic cores characterized by endothelial dysfunction, oxidative stress, and inflammation. They are a better predictor of future cardiovascular events than traditional cardiovascular risk factors in the general population.
